# Spine surgery and complication in familial dysautonomia: a case report

**DOI:** 10.3389/fsurg.2025.1559346

**Published:** 2025-06-18

**Authors:** M. Fava, G. Ciani, R. Ghermandi, C. Cini, B. Maccaferri, A. Gasbarrini

**Affiliations:** Spine Surgery Department, IRCCS Rizzoli Orthopedic Institute, Bologna, Italy

**Keywords:** dysautonomia, rare disease, spine surgery adverse events, spine surgery, spine pathology

## Abstract

Familial dysautonomia (FD) is an inherited severe congenital disease and a rare syndrome associated with progressive neuronal degeneration throughout life. Among its orthopedic conditions, FD patients have an higher incidence of kyphoscoliosis and osteomyelitis. Due to the rarity of FD and the presence of multiple comorbidities, there are currently no established guidelines for the management of vertebral pathologies associated with FD. Hence, this highlights the importance of sharing the case of our patient. The purpose of our study is to report the case of a 45-year-old patient with FD who underwent multiple spinal surgeries at our clinic, to provide possible indications for the most effective management of this rare condition.

## Introduction

Familial dysautonomia (FD), also known as Riley–Day syndrome, is an inherited severe congenital disease. It presents at birth with sensory loss, autonomic dysfunction, and early death (1). The disease was first described in 1949 by New York pediatricians Dr. Riley and Dr. Day and is now classified as hereditary sensory and autonomic neuropathy type III (HSAN-III). Only in 1970 did the FD patient registry begin, and nowadays there are only 670 genetically confirmed cases around the world (2).

As clinical care has progressed, the life expectancy of these patients has increased, and it is not uncommon for patients with FD to be treated in specialized centers (1, 2).

FD is caused by a single-point founder mutation in the ELP1 gene, located on the long arm of chromosome 9q. The mutation yields a tissue-specific skipping of exon 20 and a loss of function of the elongator-1 protein (ELP1), which is essential for the development and survival of neurons. This mutation is more common in Jews because the gene mutation arose within the Ashkenazi Jews in the sixteenth century and is present in 1:30 Jews of European ancestry. The diagnosis of FD is currently established in a proband with suggestive findings and biallelic pathogenic variants in *ELP1* (formerly *IKBKAP*) identified by molecular genetic testing (2–4).

FD is associated with neuronal degeneration progression throughout life. Considering clinical manifestation, FD-affected individuals have gastrointestinal dysfunction, autonomic crises (i.e., hypertensive vomiting attacks), recurrent pneumonia, altered pain sensitivity, altered temperature perception, anemia, and blood pressure instability. Optic neuropathy results in progressive vision loss. Older individuals often have a broad-based and ataxic gait that deteriorates over time. Developmental delay and/or intellectual disability occur in approximately 21% of individuals (1, 4).

Considering orthopedics manifestation, people with FD have a higher incidence of scoliosis, kyphoscoliosis, osteomyelitis and infections, reduced bone mineral density, and reduced trunk muscle strength. Therefore, the management of these patients is much more complex and suitable only for specialized centers (5–7).

The purpose of this study is to report the experience of a 45-year-old patient with FD who underwent multiple spinal surgeries at our clinic with a follow-up of >12 years to provide possible indications for the most effective management of these patients.

## Case report

A 45-year-old male of non-Jewish descent diagnosed with FD was admitted to our institute due to proximal junctional kyphosis (PJK) with bone loss and stenosis, which caused difficulty in walking and claudication. A thorough clinical history revealed that during his childhood, the patient had a habit of biting his fingers, leading to recurrent *Staphylococcus aureus* infections in his fingertips. This resulted in multiple amputations at his distal interphalangeal joints and tooth extractions. Additionally, he exhibited reduced tear production and lacked fungiform papillae on his tongue, both common findings in FD (8, 9). Subsequent genetic testing confirmed the diagnosis.

At the age of 20, the patient was diagnosed with acute *S. aureus* L2–L3 spondylodiscitis, which was managed with bed rest and intravenous antibiotics at another hospital. Fourteen years later, he presented with lumbar discomfort and neurological signs such as claudication. Lumbar stenosis was detected so the patient underwent further surgical treatment with the diagnosis of a new episode of spondylodiscitis. The procedure involved a decompressive laminectomy at L2–L3, followed by L2–L3 discectomy, curettage, and debridement of the vertebral bodies. Posterior spinal fusion from T12 to L4 was then performed, with a rib allograft placed in the L2–L3 disc space to aid in fusion. Prior to surgery, a CT-guided trocar biopsy of L2–L3 was conducted, confirming the diagnosis of chronic spondylodiscitis.

Following surgery, a specific rehabilitation protocol started immediately, permitting walking with the aid of a corset. The patient wore the corset consistently for 5 months post-surgery, utilizing it while sitting, standing, and walking. At the 6-month follow-up appointment after the initial operation, a computed tomography (CT) scan revealed screw mobilization, prompting a subsequent surgery for the removal of the fixation system at the T12–L4 level (10).

**Figure 1:**
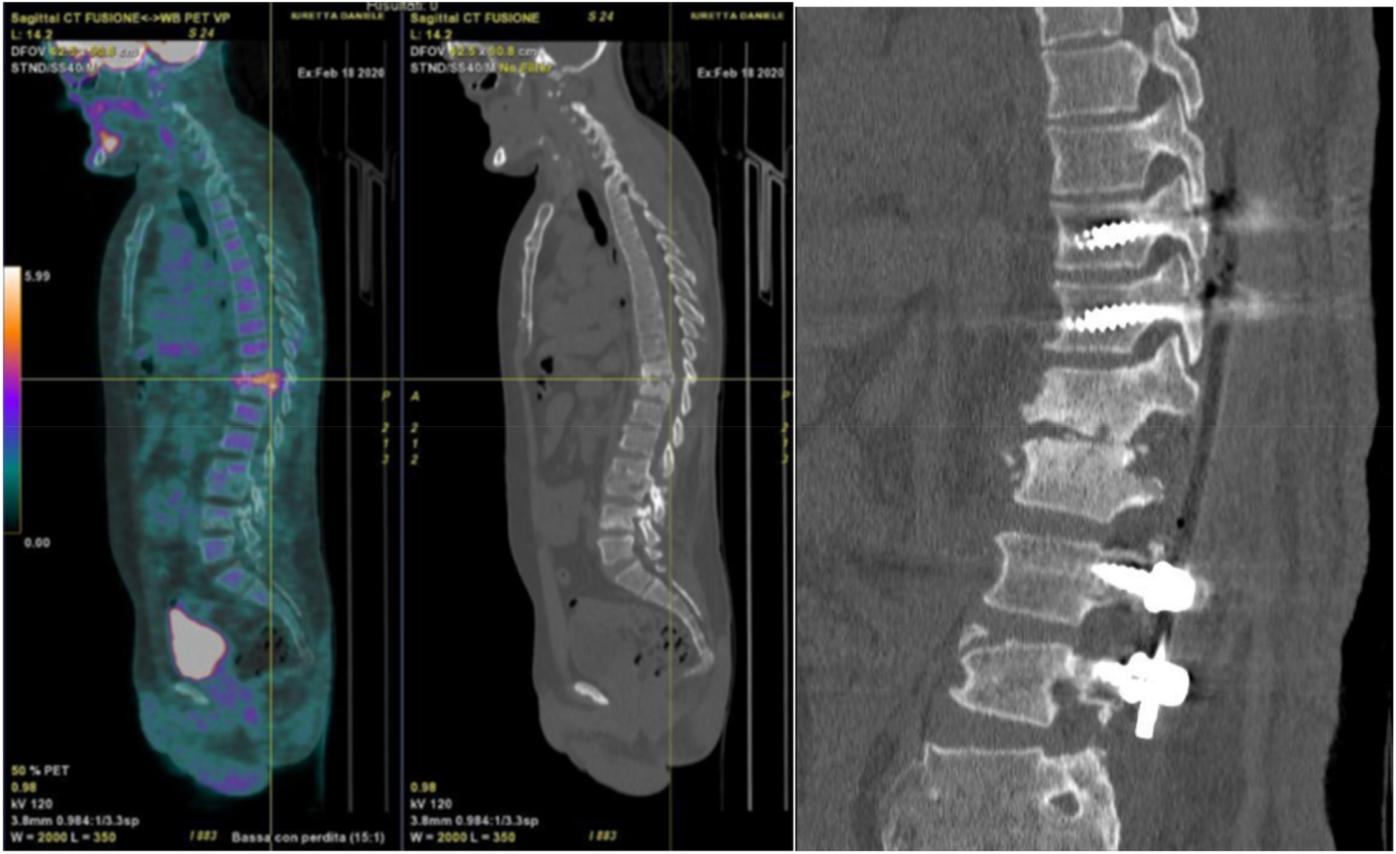
Left, PET-CT with a hyper caption of 18-FDG at the level of the infection. Right, postoperative CT showing decompression and posterior arthrodesis T10–L1 with silver-coated instrumentation.

After the latter surgery, the patient had good clinical condition. Unfortunately, 8 years later, he had worsening symptoms with evidence on CT of spondylodiscitis in T10–T11 healed into kyphosis and instability of L4–L5 due to rupture of the pars interarticularis of L4. The patient was then operated with decompression T10–L1, curettage of the lesion filled with bioglass, and stabilization T8–L1 with silver-coated instrumentation (Figure 1).

**Figure 2:**
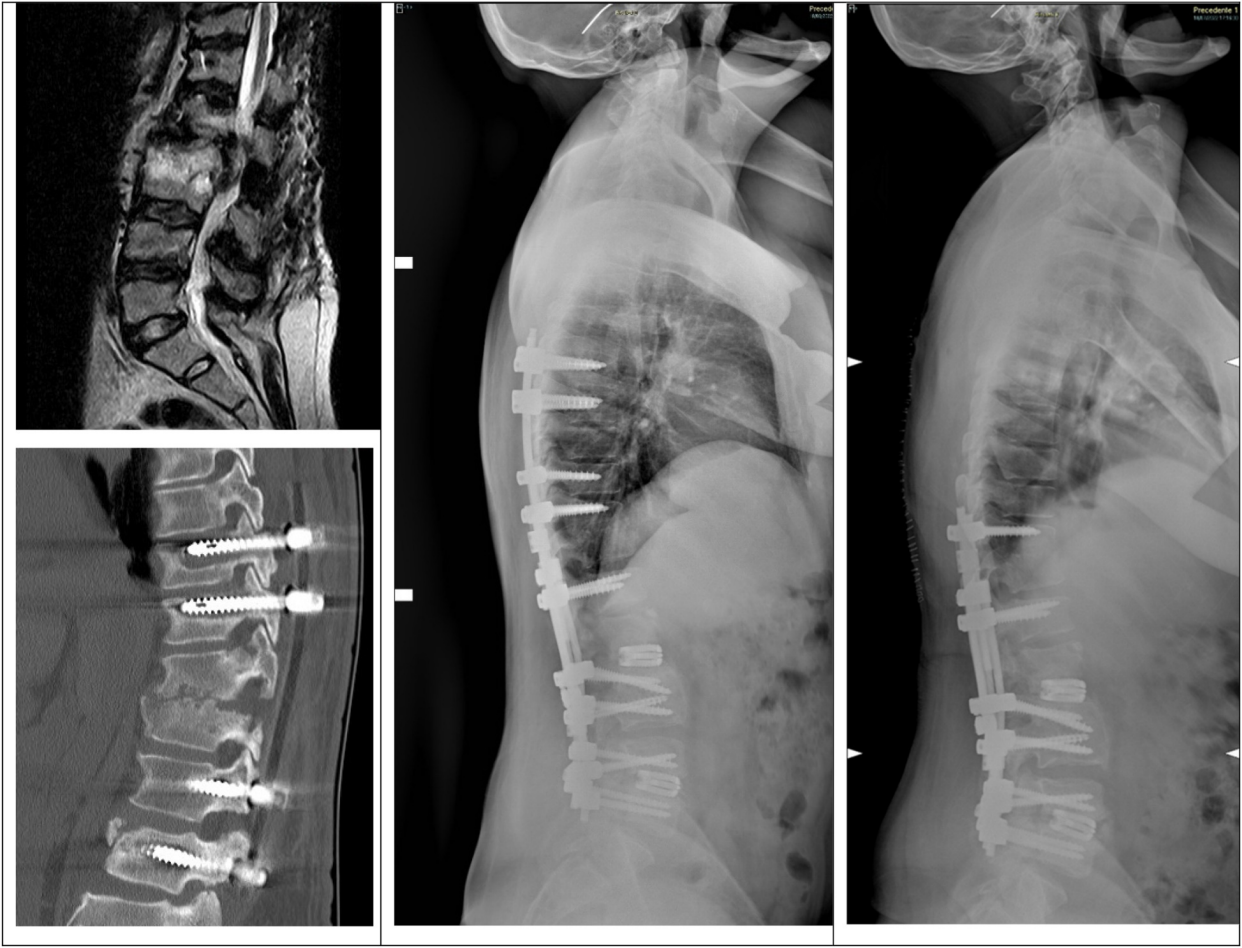
Top-left, preoperative MRI with signs of spondylodiscitis and stenosis. Bottom-left, CT showing mobilization of the screws after revision and extension T6–L2. Center, postoperative x-ray after the revision and extension of the instrumentation T6–L5 with expandable cages in L1–L2 and L4–L5. Right, postoperative x-ray after revision with shortening of the instrumentation to T10–L5.

Twenty-one months after the previous surgery, the patient presented spondylodiscitis and stenosis L1–L4 and was treated with revision of the instrumentation and extension T6–L2. Unfortunately, three weeks later, the patient went to the emergency department for persistence and worsening of low back pain. Imaging examinations showed mobilization of the distal screws, so the patient underwent revision surgery and distal extension to L5 with L1–L2 and L4–L5 expandable interbody cages. Six months later, during a follow-up visit, the imaging examination showed mobilization of the proximal screws, and the patients underwent revision surgery with shortening of the instrumentation to T10–L5 (Figure 2).

**Figure 3:**
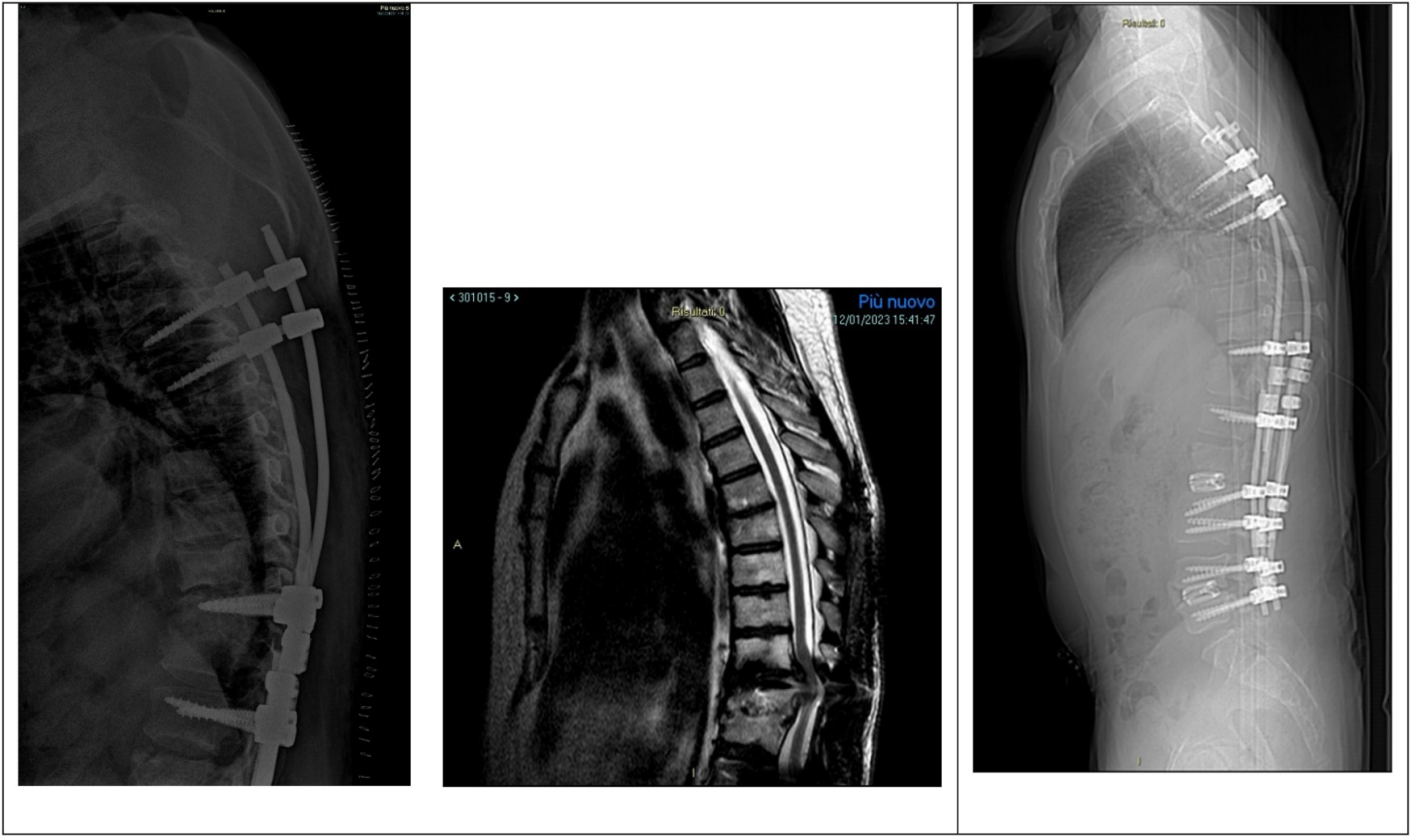
Left, mobilization of the proximal instrumentations with screws loosening on the left side. Center, MRI showing PJK with T9–T10 spinal cord compression. Right, postoperative x-ray showing proximal extension of the instrumentation to T4 and decompression T9–T10.

Six months after the latter surgery, the patient was admitted to our department for proximal junctional kyphosis (PJK) with T9–T10 spinal compression and operated with a proximal extension of the instrumentation to T4 and decompression T9–T10 (Figure 3).

One month after the latter surgery, the control PET-CT showed hyper caption of 18-FDG at T9–T10 and mobilization of the proximal screws, and the patient was treated conservatively with a cast corset and antibiotic therapy.

Two months later, due to the failure of conservative treatment with persistence of spondylodiscitis at T9–T10, mobilization of the screws, and voluminous subfascial collections, the patient underwent surgical treatment with removal of posterior instrumentation followed by antibiotic therapy and bed rest for 30 days.

**Figure 4:**
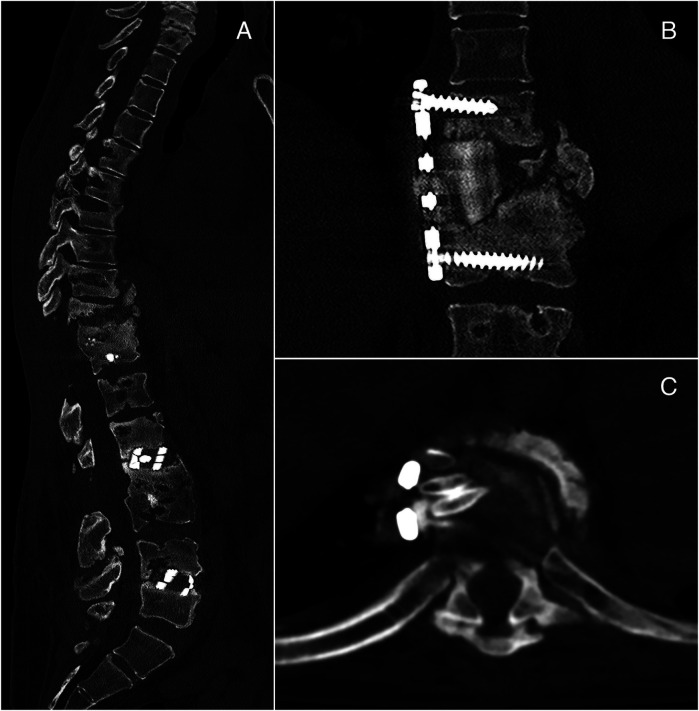
Left: thoracic compression and kyphosis at T10–T11 **(A)**. Right: postoperative CT showing dorsal arthrodesis with plate and screws and rib autograft **(B,C)**.

After 6 months from the removal of posterior instrumentations, the patient showed thoracic compression and kyphosis caused by pseudoarthrosis with vertebral collapse and instability, which were corrected with dorsal arthrodesis through thoracotomy access. Rib autograft was placed at (T10–T11) to achieve a better fixation and fill the bone loss (Figure 4). At the time of writing, 1 year and a half after the latter operation, the patient is in good clinical condition with no further complications.

## Discussion

FD, also known as Riley–Day syndrome, is a congenital disorder marked by progressive degeneration and aberrant development of the autonomic nervous system. It affects several systems, and regarding spine-related signs and symptoms, a common complication in FD patients is spinal curvature, with an incidence rate of 83%–86% in individuals aged 15–20 years (5). While scoliosis is usually the sole problem (53%–56% occurrence), kyphoscoliosis is also seen in 25%–45% of the cases (11). These spinal deformities may begin in early childhood (12).

Previous studies by Maayan et al. (7) have shown that 56% of FD patients have reduced bone density which, coupled with low physical activity levels and a low BMI, leads to a higher fracture risk (53%) (8) due to unstable gait, ataxia, and drops in blood pressure.

Piazza et al. (13) suggested that the pathogenesis of this type of arthropathy appears similar to that in other pain sensation or proprioception diseases leading to abnormal relaxation of supporting periarticular structures. This laxity results in joint instability and repetitive microtrauma, with recurrent effusions and chronic inflammation. For this mechanism, joints undergo degeneration, which leads to incongruity. Once there is incongruity, neuropathic changes ensue rapidly. Clinically, these patients present with joint instability, deformity, and weakness.

This insensitivity to pain, lack of proprioception, reduced bone density, and the tendency for joint laxity and degeneration could explain the high tendency of mobilization of the instrumentation that we reported in our patient.

Regarding recurrent infections, Klebanoff et al. (14) reported a case of familial dysautonomia associated with recurrent osteomyelitis in a non-Jewish girl, but the reason for these increased infections does not appear to be clear, and there are no studies in the literature. Huneycutt et al. (15) suggested that patients with familial dysautonomia are not at a particularly increased risk of infectious diseases. However, when these patients develop an infectious process, they may not have the usual symptoms of infection (16). Some infections may be silent and only manifest as fever or by a dysautonomic crisis. Some reports suggested that the occurrence of osteomyelitis was associated with spinal deformities and Charcot joints (17). However, there is no evidence that osteomyelitis occurs more frequently in patients with familial dysautonomia (18).

In our experience, the patient was polyallergic, and this made the management of antibiotic therapy difficult. However, he has no nickel allergy, and even the use of nickel-free instrumentation apparently did not reduce postoperative complications.

Treatment of this rare disease is currently symptomatic and preventive, and for this reason, there is a need for multi-specialty management for these patients. Regarding the symptoms of the musculoskeletal system, there are currently no guidelines. Piazza et al. (13) suggested that the primary goals of treatment are vertebral stabilization and prevention of pathologic motion. Indications for surgery include failure of nonoperative treatment with progressive instability, deformity, or neurologic lesion. For the authors, spinal decompression alone has had no significant effects on neurologic status, and in all likelihood, it predisposes to further instability in the future; instead, rigid internal fixation as well as anterior spinal fusion may be required in addition to standard posterior fusion techniques. Bar-On et al. (19) suggested that bracing is of questionable benefit, surgical intervention should be considered once curve progression is well documented, and arthrodesis should be extended as far proximally as possible to prevent junctional kyphosis. Furthermore, according to González-Duarte et al. (2), bracing for spinal deformities can cause inadvertent pressure ulcers and inhibit chest wall movements.

## Conclusion

Based on our experience, surgical treatment for patients with FD should be considered only when preventive conservative approaches are found to be insufficient. When surgery is needed, it is important to be aware that these patients have a higher risk of mechanical complications and infections. Thus, in our experience, the surgical approach in patients with FD is designed to ensure primary stability through the shot area of arthrodesis and possibly even with double access. However, studies on patients by primary care are essential, knowing the high risk of systemic infection, spinal and extremity deformities that can be created, and thus the need for specialized evaluation.

## Data Availability

The raw data supporting the conclusions of this article will be made available by the authors, without undue reservation.
